# Bioprocess development for microbial production and purification of cellobiose lipids by the smut fungus *Ustilago maydis* DSM 4500

**DOI:** 10.1007/s00449-025-03127-3

**Published:** 2025-01-10

**Authors:** André D. Valkenburg, George M. Teke, Eugéne van Rensburg, Robert W. M. Pott

**Affiliations:** Department of Chemical Engineering, Stellenbosch University, Private Bag X1, Matieland, 7602 South Africa

**Keywords:** Biosurfactants, Glycolipids, Cellobiose lipids, Upstream production, *Ustilago maydis*

## Abstract

Cellobiose lipids (CBLs) are a class of glycolipid biosurfactants produced by various fungal strains. These compounds have gained significant interest due to their surface-active and antifungal properties, which are comparable to traditional synthetic surfactants and antimicrobials. Despite their potential applicability in various cosmetic, pharmaceutical, and agricultural formulations, significantly less research has been focused on their production and purification in comparison to other glycolipid biosurfactants, such as mannosylerythritol lipids (MELs) and sophorolipids. Hence, this work proposes the development of a bioprocess that involves the microbial production and high-level chromatographic purification of CBLs from a submerged culture of *Ustilago maydis* DSM 4500. After a highly purified CBL product was obtained, the factors affecting the production of this glycolipid were investigated. It was demonstrated that *U. maydis* DSM 4500 produces a specific structural variant of CBLs at a concentration of 1.36 g/L on an optimized the growth medium. Also, it was established that when the C/N ratio was decreased, the CBL titer increased by 2.3-fold. Furthermore, supplementing the culture with ZnSO_4_ at a concentration of 0.04 mg/L further increased CBL concentration to 4.95 g/L, representing the highest CBL titer achieved in a single-stage bioprocess to date. This study developed a methodology for utilizing *U. maydis* as a high-level CBL producer, which could challenge other familiar CBL producers, such as *Sporisorium scitamineum* and *Cryptococcus humicola*.

**Supplementary Information:**

The online version contains supplementary material available at 10.1007/s00449-025-03127-3.

## Introduction

Surfactants are surface-active compounds with the ability to decrease the surface tension at liquid–gas interfaces, as well as the interfacial tension between immiscible liquid phases in emulsions. Due to these properties, they have critical applications across various sectors, including the oil and gas, pharmaceutical, and cosmetic industries [1–3]. For example, due to their amphiphilic nature, surfactants are extensively used as detergents in industrial cleaning products, as well as de-emulsifiers for the recovery of oils during bioremediation [4]. Although synthetic surfactants are useful in various applications, they are commonly non-biodegradable and toxic to the environment, especially to aquatic ecosystems. In addition to this, their production relies on the utilization of non-renewable petroleum-based sources [5–7]. Consequently, alternatives to synthetic surfactants are being sought after, with biosurfactants being extensively studied for their potential improved environmental properties [8].

Biosurfactants are naturally produced by various living organisms, including plants, animals, bacteria, fungi, and yeast [9]. Their microbial production is particularly interesting from an industrial perspective due to these fermentative processes' mild operating conditions and lower carbon footprint [10]. Five categories of microbial biosurfactants have been defined based on the structure of their hydrophilic head groups, including glycolipids, lipopeptides, particulate biosurfactants, polymeric biosurfactants, and phospholipids [10, 11]. Glycolipids are a class of low molecular weight biosurfactants that consist of long-chain aliphatic or hydroxyl aliphatic acids linked to mono-, di-, tri- and tetra-saccharide hydrophilic sugars [12]. This class of microbial biosurfactants has gained recognition due to their relatively high production yields, as well as their applicability in pharmaceutical and cosmetic formulations [13, 14].

Focusing on glycolipid biosurfactants, cellobiose lipids (CBLs) are an understudied biosurfactant, which consist of a hydrophilic cellobiose core attached to a hydrophobic long-chain fatty acid residue. CBLs have gained interest due to their interesting gelling characteristics and antifungal activity against a wide range of phytopathogenic fungal strains [15, 16]. These properties render CBLs ideal for producing novel biomaterials, including ointments and creams with antifungal activity, which can be used to control pathogenic fungi in the agricultural and medical industries. Furthermore, the surface-active properties of CBLs are attractive for the formulation of cosmetic and industrial cleaning products [17]. However, research on the applicability of CBLs remains limited due to the relative paucity of research focused on developing a process for the microbial production and purification of these compounds.

The secretion of CBLs by various strains of dimorphic fungi grown on mono- and disaccharide sugars is well described [17, 18]. Morita et al. [19] investigated the production of CBLs by *Cryptococcus humicola* JCM 1461 from a production medium supplemented with glucose as the primary carbon source, using both a single-batch and fed-batch approach [19]. Similarly, Oraby et al. [17] reported the production of CBLs by *Sporisorium scitamineum* DSM 11941 grown on a production medium containing sucrose as the main carbon source [17]. This titer could again be significantly increased by implementing a fed-batch approach with a production medium containing glucose as the primary carbon source. However, low CBL product titers (in the range of 5 g/L to 15 g/L) in comparison to other glycolipid biosurfactants (often exceeding 100 g/L) remain the biggest limiting factor for the commercial adoption of CBLs [20, 21]. Therefore, to develop a scalable bioprocess capable of producing CBLs at the required titers for industrial applications, there is a need to investigate the factors affecting the production of CBLs from different producing organisms.

*Ustilago maydis*, a dimorphic fungus of the family *Ustilaginaceae*, that causes smut disease in corn, was the first identified CBL producer and served as the model organism for research focused on the CBL biosynthetic pathways [22–24]. However, despite the importance of this organism as a plant pathogen, past research focused mostly on the metabolism and characterization of CBLs, whereas no research has been focused on developing a bioprocess for the production of CBLs by *U. maydis*. Therefore, a bioprocess for the efficient production of the structural variants of CBLs produced by this strain has not been established. In addition to this, although *U. maydis* has been established as a potential high-level CBL producer, it is not understood how effectively this strain can produce CBLs compared to other high-level CBL-producing microbes, such as *Cr. humicola* and *S. scitamineum*. To develop a bioprocess for the effective commercial production of CBLs, it is imperative to understand which strains produce CBLs at the highest efficiency. Therefore, this study developed a bioprocess for the microbial production and purification of CBLs by *U. maydis* DSM 4500. Hence, the study proposes a novel route toward obtaining a highly pure CBL extract, while providing an improved understanding of the factors affecting CBL production by *U. maydis*. As a result, this solidifies the potential of developing a scalable bioprocess to produce CBL, centered around *U. maydis* as opposed to other high-level CBL producers.

## Materials and methods

### Chemicals and equipment

Malt extract, bacteriological agar, yeast extract, D-(+)-glucose (BioReagent, purity 99%), sodium nitrate (purity 99%), magnesium sulfate (purity 98%), monobasic potassium phosphate (purity 99%), copper (II) sulfate pentahydrate (purity 98%), manganese (II) sulfate monohydrate (purity 98%), iron (III) chloride hexahydrate (purity 98%), zinc sulfate heptahydrate (purity 98%), calcium chloride hexahydrate (purity 98%), cobalt (II) chloride hexahydrate (purity 98%), ethyl acetate (purity 99.5%), chloroform (purity 99.8%), methanol (purity 99.9%), ethanol (purity 99%), ammonium hydroxide (ACS reagent, 28.0–30.0% NH_3_ basis), p-anisaldehyde (98%), acetic acid (glacial, 100%), and hydrochloric acid (purity 37%) were all obtained from Sigma-Aldrich, South Africa.

### Culture conditions

#### Microorganisms

*Ustilago maydis* DSM 4500 was obtained from the German Collection of Microorganisms and Cell Cultures (DSMZ) in Braunschweig, Germany. Stock cultures were cultivated on agar plates consisting of (per liter): 30 g malt extract, 5 g mycological peptone and 15 g bacteriological agar. The agar plates were incubated at 30 °C for 4 days, before being stored at 4 °C. The stock cultures were renewed every two weeks onto fresh agar plates.

#### Seed cultures

Seed cultures were prepared by inoculating stock cultures into 250 mL baffled shake flasks containing 50 mL of a synthetic medium consisting of (per liter): 40 g glucose, 3 g NaNO_3_, 0.3 g MgSO_4_, 0.3 g KH_2_PO_4_, and 1 g yeast extract [25]. Before inoculation, the shake flasks and synthetic medium were sterilized in a autoclave at 121 °C for 15 min. The starter cultures were incubated on an orbital shaker adjusted to 150 rpm at 30 °C for 3 days [26].

#### Microbial production of a cellobiose lipids

CBLs were produced by inoculating 1 mL of seed culture into 500 mL baffled shake flasks containing 100 mL of a defined CBL production medium. The medium consisted of (per liter): 50 g glucose, 0.6 g NaNO_3_, 0.5 g MgSO_4_, 0.1 g NaCl and 0.1 g CaCl_2_ [17]. The pH of the culture was adjusted to 2.6 through the addition of a 1.0 M HCl solution, and controlled with a phosphate-citrate buffer, which consisted of (per liter): 1.71 g citric acid and 0.31 g Na_2_HPO_4_. The cultures were incubated on a rotary shaker (150 rpm) at 30 °C for 10 days [17].

#### Investigating the factors affecting CBL production

Three factors that affect the production of CBLs have been identified: the carbon-to-nitrogen (C/N) ratio, the pH, and the addition of trace elements in the synthetic medium [17]. Different C/N ratios were considered, including 16.67 g glucose/g NaNO_3_, 83.33 g glucose/g NaNO_3_ and 166.67 g glucose/g NaNO_3_, which were achieved through the addition of 3 g, 0.6 g and 0.3 g of NaNO_3_ (per liter) to the prepared growth medium, respectively.

For the pH investigation, three pH values (2.6, 4.0 and 6.0) were investigated. The pH of the medium was adjusted by adding HCl and NaOH solutions of 1.0 M, respectively. To control the pH at 4.0, a citrate–phosphate buffer system was used which consisted of (per liter): 1.18 g citric acid and 1.09 g Na_2_HPO_4_ [27]. Arnstein et al. [27]The pH was controlled at 6.0 through the addition of (per liter) 1 g KH_2_PO_4_ [25].

To investigate the effect of trace elements on the production of CBL, the growth medium was individually supplemented with literCuSO_4_, MnSO_4_, FeCl_3_, ZnSO_4_, CoCl_2_, and Na_2_MoO_4_ at a concentration of 0.04 mg/L, as shown in literature for the production of glycolipids [17, 28].

### Isolation and purification of cellobiose lipid standard

#### Extraction of CBLs from the liquid culture

After the 10-day cultivation, the cultures were centrifuged at 13.0 g for 5 min. The pellet containing the biomass and CBL crystals was resuspended in acidic water (pH 2) to remove residual sugars. The biomass and CBLs were collected by centrifugation at 13.0 g for 5 min. CBLs were dissolved by resuspending the pellet in ethanol at an equal volume to the original culture sample. The biomass was then collected by centrifugation at 13.0 g for 5 min. The ethanol was then allowed to evaporate under a vacuum to yield a crude glycolipid extract. This extract was suspended and centrifuged twice in 4ml ethyl acetate per gram extract to remove residual MELs. The suspension was centrifuged at 13.0 g for 10 min, and the resulting pellet was dried to yield a CBL extract [17].

#### Purification of CBL standard by thin layer chromatography (TLC)

The CBL extract was dissolved in ethanol at a concentration of 50 g/L and spotted onto a silica TLC plate (Gel 60 F254, Merck, Darmstadt, Germany) and developed with a mobile phase which consisted of a mixture of 65 mL chloroform, 15 mL methanol and 2 mL ammonium hydroxide, respectively [29]. An anisaldehyde solution was prepared by adding 0.5 mL of anisaldehyde to 10 mL of glacial acetic acid, before adding 85 mL of methanol and 5 mL of concentrated sulfuric acid, in that order. The TLC plates were then lightly sprayed with an anisaldehyde solution and heated to 110 °C for 5 min on a heating plate. CBLs and MELs appeared as purple spots on the developed TLC plates [30].

To produce a purified CBL standard, the silica which was at the same height as the CBL spots on subsequent TLC plates which were not sprayed with the anisaldehyde solution, was scraped off and suspended in methanol to extract the purified CBLs from the silica. The silica was removed by centrifuging the suspension at 13.0 g for 10 min, filtered through a 0.22 μm nylon filter and evaporated to yield a purified CBL extract. After the structure of the purified CBL extract was confirmed by ESI–MS and NMR analysis, the extract was used as a standard for the quantification of CBLs in subsequent culture media using high-performance liquid chromatography (HPLC).

### Analytical methods

#### Microscope imaging

Samples from the liquid cultures were dropped on microscopic slides and observed through a Zeiss AxioStar Plus binocular microscope at 10 ×, 20 ×, and 40 × magnification. Images were taken through the eyepiece of the microscope.

#### Structural analysis of CBL standard

##### Nuclear magnetic resonance (NMR)

The purified MEL and CBL extracts were dissolved in methanol-d_6_ and subject NMR analysis to determine their molecular structures. ^1^H NMR and ^13^C NMR spectra were recorded at 25 °C on a 600 MHz Bruker NEO NMR spectrometer utilizing a 5 mm TBO i-probe. The default 30° ^1^H and ^13^C pulse sequences, available from the Topspin 4.3 software package, were employed using a 30° excitation pulse angle. The recorded spectra were processed on a licensed Mestrenova 12 software package. Manual integration and peak picking was performed following chemical shift referencing on the residual methanol signal. The recording and processing of the data were performed by the Central Analytical Facilities (CAF) at Stellenbosch University.

##### Electrospray ionization–mass spectrometry (ESI–MS)

ESI–MS analysis was employed to determine the molecular weight of the purified glycolipid extracts. The analysis was performed by the Central Analytical Facilities (CAF) at Stellenbosch University. Samples were dissolved in methanol and directly injected into a Waters Synapt G2 coupled to a Waters UPLC and an ESI probe. The analysis was performed in negative ion mode and the sampling cone voltage was set to 15 V.

#### Analysis of the liquid culture constituents

##### UV spectroscopy

The concentration of biomass in the liquid cultures was determined by measuring the OD of samples taken from the cultures in a UV spectrophotometer at a wavelength of 600 nm. The cell concentration was then calculated from a previously constructed calibration curve.

##### HPLC analysis

The concentration of carbon source in the medium was measured with a Dionex Ultimate 3000 HPLC System equipped with RI detector. The system was equipped with a Biorad Aminex HPX-87H column (8 × 300 mm). The quantification was performed at a temperature of 65 °C with 5 mM H_2_SO_4_ at a flowrate of 0.6 mL/min as the eluent [30].

The concentration of the nitrates in the medium was measured with a Dionex Aquion System equipped with a suppressed conductivity detector. The system was equipped with a Dionex IonPac AS4A-SC column (4.6 × 250 mm). The quantification was performed at room temperature with a mixture of 1.8 mM sodium carbonate and 1.7 mM Bicarbonate at a flowrate of 1 mL/min as the eluent [30].

To measure the concentration of CBLs in the medium, samples from the liquid cultures were diluted with ethanol at a ratio of 1:1 to ensure that all of the glycolipids were in solution. The quantification of CBLs could then be performed by subjecting these samples to HPLC analysis on a silica-gel column (RPSep PRX-1, 4.6 × 150 mm) with a low-temperature evaporative light scattering detector using a gradient solvent program consisting of various proportions of methanol and chloroform (from 100:0 to 0:100, v/v) at a flow rate of 1 mL/min [30].

## Results and discussion

### Production and identification of CBLs by *U. maydis*

Despite the interest in CBL production and application, there is a lack of a commercially available standard that can be used to quantify CBLs in solution. Therefore, this section describes the production of a purified CBL standard from *U. maydis*, as well as the subsequent ESI–MS and NMR analyses implemented to analyze the structure of the CBL standard.

#### Production of CBLs from glucose by *U. maydis*

**Fig. 1:**
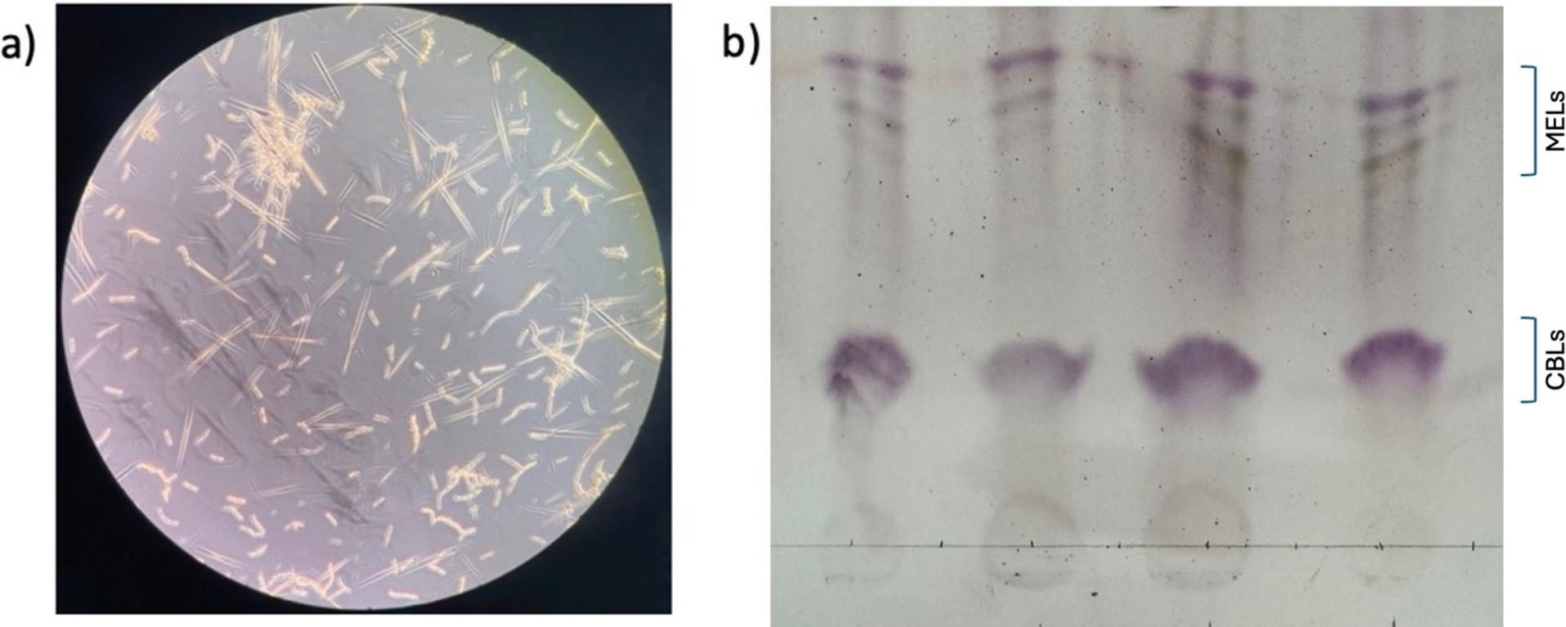
**a** A microscopic image (40 × magnification) of the *U. maydis* DSM 4500 cultures showed the formation of needle-like crystals which indicated the presence of CBLs. **b** The retention factors of compounds extracted from the supernatant of submerged *U. maydis* DSM 4500 culture

The microscopic images presented in Fig. 1a show the formation of needle-like crystals when the *U. maydis* cultures were grown on the defined CBL production medium, presented in “Microbial production of a cellobiose lipids” section. This crystal formation was consistent with the findings of Oraby et al. [17], when *S. scitamineum* DSM 11941 culture was grown on a similar medium. After implementing the glycolipid extraction procedure presented in “Extraction of CBLs from the liquid culture” section the developed TLC plates of the crude CBL extracts are shown in Fig. 1b, where the stained spots corresponded to six retention factors emanating from the product mixture. The four spots with the highest retention factor (R_f_) ranging between 0.85, and 0.63 were consistent with those reported for MEL-A, MEL-B, MEL-C, and MEL-D. The intensity of the spots suggested that the MELs had a weaker interaction with the stationary phase in comparison to CBLs [31]. The two spots with R_f_ values of 0.34 and 0.27 (Fig. 1b) were consistent with CBLs, as reported by [17]. Therefore, the silica on subsequent TLC plates corresponding to the height of these spots was scraped off and extracted with methanol. These extracts were then analyzed using ESI–MS and NMR to verify the structure of the CBLs produced by *U. maydis* DSM 4500.

#### Structural analysis of the produced CBL homologs

##### ESI–MS analysis

The isolated CBLs were analyzed by ESI–MS in negative ion mode and a dominant ion mass of [M-H]^−^ at m/z 783.4, as presented in Fig. 10 (Supplementary data). This observation was consistent with the monoisotopic molecular weight of *ustilagic acid-B* with a molecular formula of C_36_H_63_O_18_. The deprotonated negative monoisotopic molecular ion of the CBL variant was calculated as 783.4 g/mol.

##### NMR analysis

**Fig. 2:**
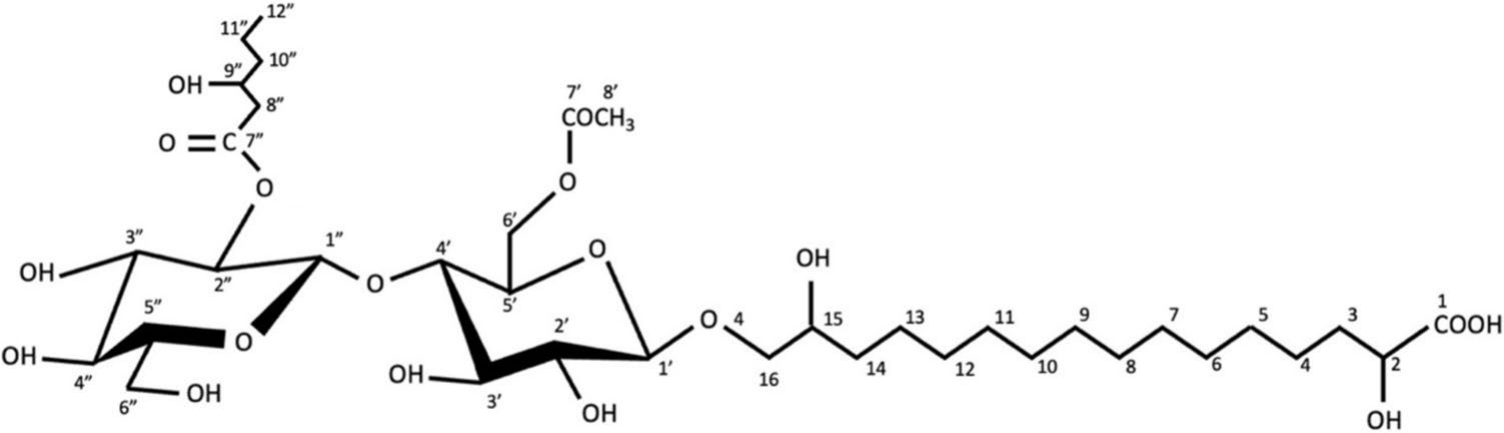
The proposed chemical structure of the CBLs isolated from the *U. maydis* DSM 4500 culture

To confirm the proposed structure of *ustilagic acid B* as suggested by mass spectrometry, the purified CBL extract was subject to ^1^H and ^13^C NMR analysis with their spectra presented in Fig. 8 and Fig. 9 (Supplementary data), respectively. To confirm the proposed structure of ustilagic acid B from mass spectrometry, the purified CBL extract was subject to ^1^H and ^13^C NMR analysis with their plots presented in Fig. 8 and Fig. 9 (Supplementary data). The spectra were then compared to those obtained for CBL-B, or 2-O-3-Hydroxyoctanoil-3-O-acetyl-β-D-glucopyranosyl-(1–4)-6-O-acetyl-β-D-glucopyranosyl-(1–16)-3,15,16-trihydroxyhexodecanoic acid, by Yang et al. [32], as shown in Table 1 [32]. It was determined that the NMR data aligned with the published data, indicating that isolated CBLs are identical to those described by Yang et al. [32] (Fig. 2).

**Table 1:** The ^1^H and ^13^C NMR data observed for the purified CBL extract compared to those recorded for CBL-B, as reported by Yang et al. [32]

Carbon	1H—reported by Yang et al. [32]		1H—observed		∆ ppm	13C—reported by Yang et al. [32]	13C—observed	∆ ppm
	ppm	Multiplicity	ppm	Multiplicity		ppm	ppm	
1	1.62	m	1.62	N/A		179.3	179.3	0
2	3.74	m	3.76	ddt	− 0.02	72.8	72.93	− 0.13
3	1.62 and 1.74	m and m	1.63 and 1.74	ddq and td	− 0.01 and 0	36.6	36.69	− 0.09
4	1.43	m	1.45	m	− 0.02	27.3	27.39	− 0.09
5	1.29	m	1.32	m	− 0.03	31.9–31.6	32.06	− 0.16
6							32.01	N/A
7							32	
8							31.97	
9							31.96	
10							31.93	
11							31.88	
12							31.72	− 0.12
13	1.43	m	1.45	m	− 0.02	27.7	27.84	− 0.14
14	1.74	m	1.74	N/A	0	35.4	35.53	− 0.13
15	3.37	m	3.37	m	0	72.5	72.65	− 0.15
16	3.36	m	3.36	N/A	0	76.9	77.03	− 0.13
1'	4.31	d	4.31	N/A	0	105.7	105.83	− 0.13
2'	3.25	m	3.27	m	− 0.02	75.9	76.07	− 0.17
3'	3.36	m	3.36	N/A	0	77.3	77.46	− 0.16
4'	3.5	m	3.52	m	− 0.02	82.5	82.64	− 0.14
5'	3.57	m	3.58	m	− 0.01	74.7	74.86	− 0.16
6'	4.1 and 4.32	m and m	4.11 and 4.32	m and m	− 0.01 and 0	65.4	65.5	− 0.1
7'	N/A	N/A	N/A	N/A	N/A	173.6	173.74	− 0.14
8'	2.03	s	2.08	s	− 0.05	21.9	22.02	− 0.12
1"	4.55	d	4.56	d	− 0.01	103.3	103.46	− 0.16
2"	4.77	m	4.77	dd	0	76.5	76.65	− 0.15
3"	3.35	m	3.35	N/A	0	77.1	77.23	− 0.13
4"	4.09	m	4.09	N/A	0	72.6	72.72	− 0.12
5"	3.37	m	3.37	m	0	79.4	79.61	− 0.21
6"	3.66 and 3.9	m and m	3.67 and 3.91	m and m	− 0.01 and − 0.01	63.5	63.63	− 0.13
7"	N/A	N/A	N/A	N/A	N/A	173.8	173.95	− 0.15
8"	2.54	m	2.55	ddd	− 0.01	44.3	44.48	− 0.18
9"	4.01	m	4.02	tt	− 0.01	68.2	70.21	− 2.01
10"	1.43	m	1.45	m	− 0.02	41.4	41.55	− 0.15
11"	1.43	m	1.45	m	− 0.02	20.9	21.01	− 0.11
12"	0.94	t	0.94	dt	0	15.5	15.62	− 0.12

### Factors affecting the production of CBLs

#### Investigating the time course of CBL production

**Fig. 3:**
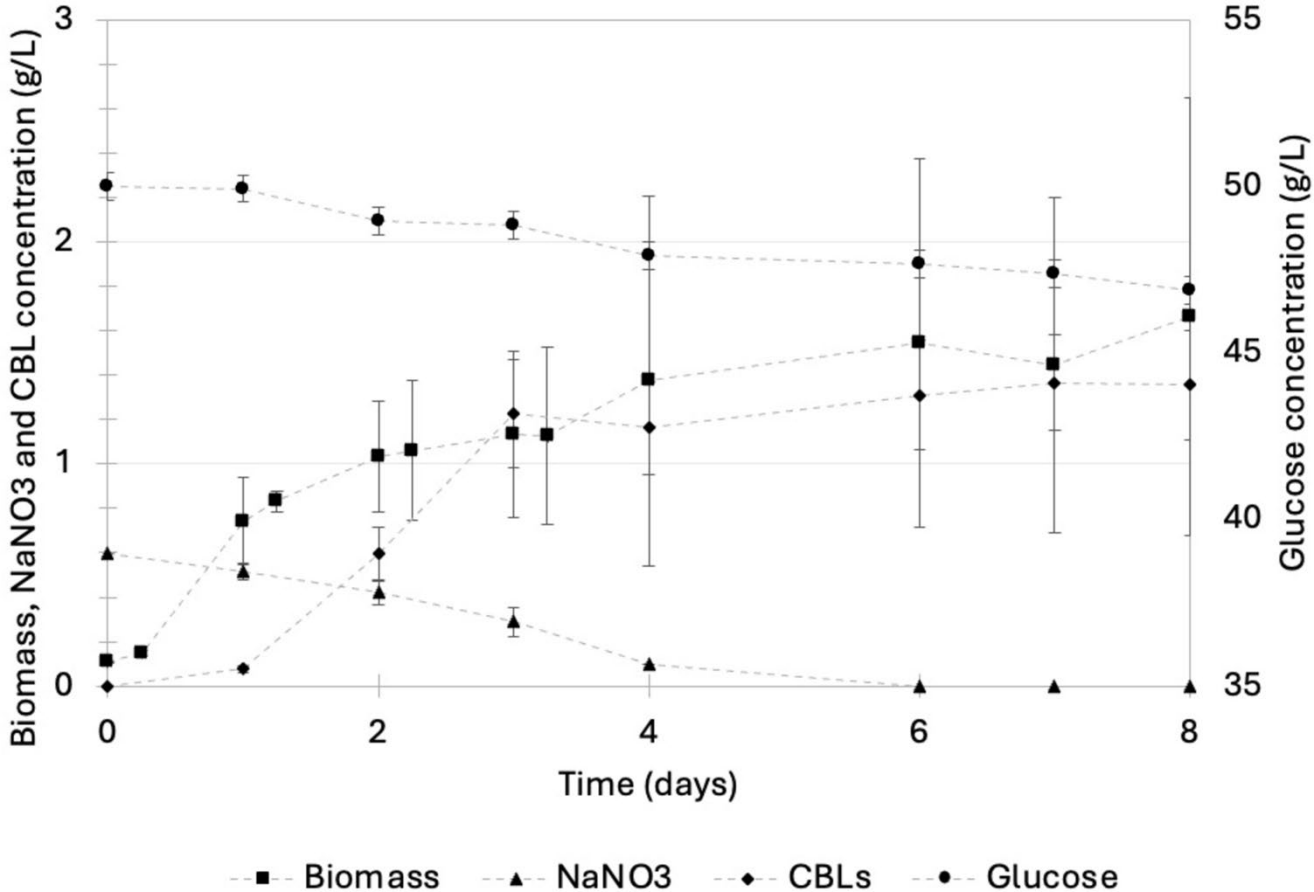
A growth curve depicting the biomass formation, substrate consumption, and CBL production by *U. maydis* DSM 4500. The production medium contained 50 g/L glucose and 0.6 g/L NaNO_3_ as the sole carbon and nitrogen sources, respectively. This represented a C/N ratio of 83.33 g glucose/g NaNO_3_. The pH of the production medium was controlled at a pH of 2.6. The error bars represent the standard deviation of triplicate experiments

To gain an improved understanding of the dynamics involved in the production of CBLs, the production procedure discussed in “Microbial production of a cellobiose lipids” section was implemented and the concentration of biomass, nutrients, and CBLs in the culture medium was monitored over 8 days (Fig. 3). The stationary phase coincided with nitrogen depletion after approximately 6 days where a biomass concentration of 1.66 g/L was recorded. CBL production occurred primarily during the early growth phase, reaching a concentration of 1.31 g/L at the onset of the stationary growth phase. There was no marked increase in CBL after the stationary growth phase was reached in spite of the presence of 45 g/L glucose. These results suggested that CBL production is growth-associated and does not require nitrogen starvation in excess glucose, as is the case with MELs, as previously reported [30]. Furthermore, the significant amount of unconsumed glucose at the onset of the stationary phase suggests that the initial glucose concentration in the medium could be significantly decreased. This should be explored in future work focusing on optimizing medium composition for CBL production.

#### The effect of C/N ratio on the production of CBLs

**Fig. 4:**
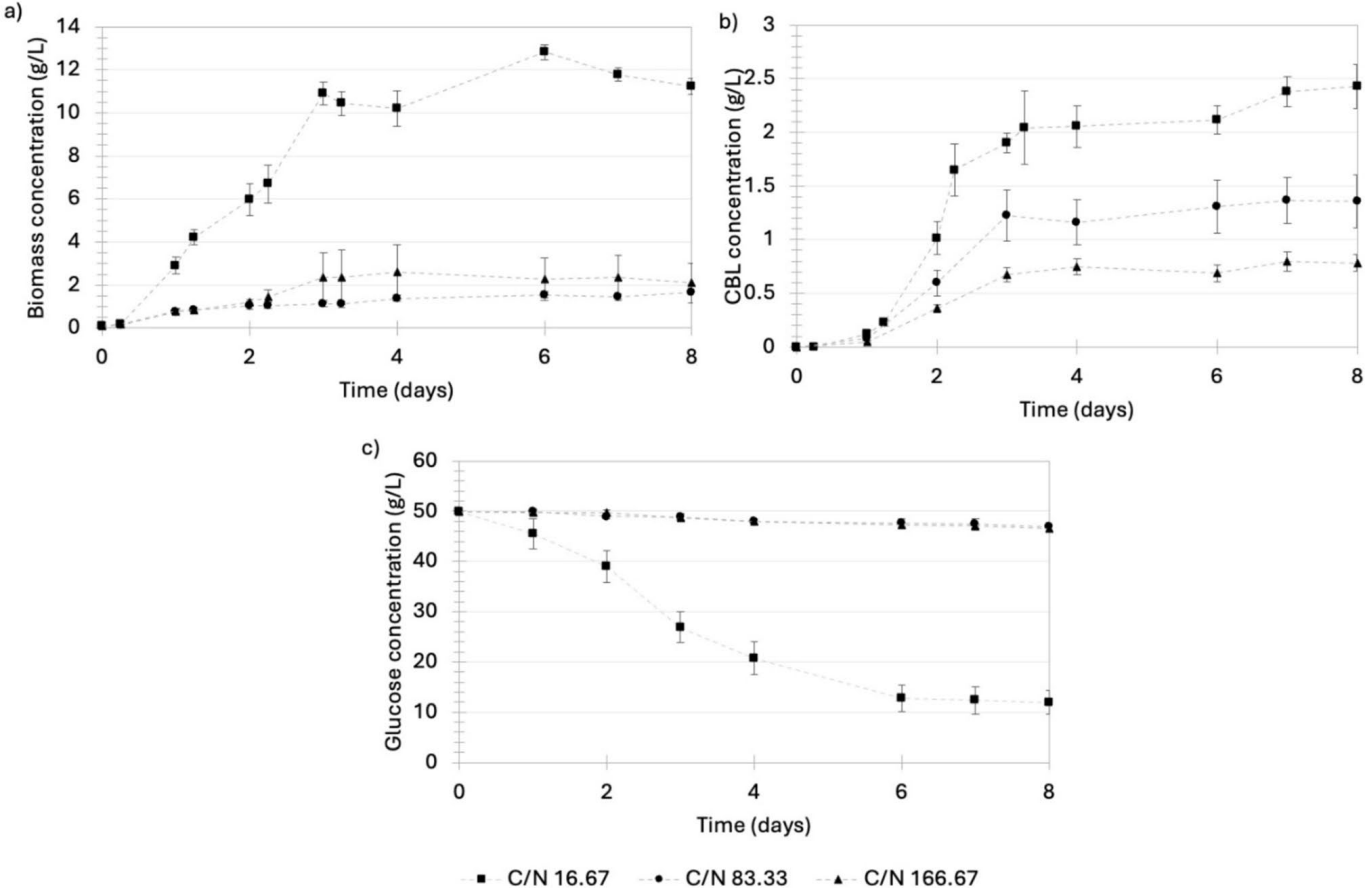
**a** The microbial growth, **b** CBL production, and **c** glucose consumption by *U. maydis* DSM 4500 at a C/N ratio of 40 g glucose/g NaNO_3_, 200 g glucose/g NaNO_3_, and 400 g glucose/g NaNO_3_. The pH of the production medium was controlled at a pH of 2.6. The error bars represent the standard deviation of triplicate experiments

Three different C/N ratios of 40 g glucose/g NaNO_3_, 200 g glucose/g NaNO_3_, and 400 g glucose/g NaNO_3_ were used to determine the effect of nitrogen on CBL production by *U. maydis* (Fig. 4). It was observed that biomass formation decreased significantly when the C/N ratio in the medium was increased, as shown in Fig. 4a. At a C/N ratio of 16.67 g glucose/g NaNO_3_, a maximum biomass concentration of 12.81 g/L was achieved with a further decrease to 1.54 g/L and 2.27 g/L when the C/N ratio was increased to 83.33 g glucose/g NaNO_3_ and 166.67 g glucose/g NaNO_3_, respectively. These results indicated that nitrogen plays a crucial role in the growth of *U. maydis*. Also, it was observed that, under all C/N ratios conditions, primary CBL formation occurred during the early growth stage (from inoculation until approximately day 2), as shown in Fig. 4b. Furthermore, there were no significant increases in the CBL titers after nitrogen starvation was achieved on day 6. The highest product titer of 2.43 g/L was achieved at a C/N ratio of 16.67 g glucose/g NaNO_3_. However, an increase in the C/N ratio to 83.33 g glucose/g NaNO_3_ or 166.67 g glucose/g NaNO_3_ led to a reduced product titer of 1.36 g/L and 0.78 g/L, respectively. Since it was established that CBL production by *U. maydis* DSM 4500 is highly growth-associated, the decrease in product titer at higher C/N ratios was attributed to the significant decrease in biomass formation, as shown in Fig. 4a. However, to understand how effectively individual cells produced CBLs at different C/N ratios, the yield of CBLs per biomass (Y_P/X_) and substrate consumed (Y_P/S_) was determined, as shown in Table 2.

**Table 2:** The average biomass and CBL titers, yield and rates of formation by *U. maydis* DSM 4500 at pH 2.6 and C/N ratios of 16.67 g glucose/g NaNO_3_, 83.33 g glucose/g NaNO_3_ and 166.67 g glucose/g NaNO_3_

C/N (g/g)	Titres (g/L)		Yield (g/g)					Rates (g/L.h)	
	Biomass	CBLs	Biomass per glucose consumed	Biomass per nitrogen consumed	CBLs per glucose consumed	CBLs per nitrogen consumed	Product per biomass	Biomass	CBLs
16.67	11.212	2.426	0.295	3.737	0.064	0.809	0.216	0.058	0.013
83.33	1.661	1.356	0.534	2.769	0.436	2.260	0.816	0.009	0.007
166.67	2.104	0.783	0.616	7.014	0.229	2.609	0.372	0.011	0.004

Although product titers were maximized at a C/N ratio of 16.67 g glucose/g NaNO_3_, *U. maydis* DSM 4500 could produce CBLs more efficiently at higher C/N ratios in terms of the product yield per biomass formation and glucose consumption, as shown in Table 2. The gram CBLs produced per gram biomass produced was shown to increase from 0.216 g/g at a C/N ratio of 16.67 g glucose/g NaNO_3_, to 0.816 g/g at a C/N ratio of 83.33 g glucose/g NaNO_3_. When the C/N ratio was further increased to 166.67 g glucose/g NaNO_3_, the biomass yield decreased to 0.372 g/g. A similar trend was observed in the substrate yields (*Y*_P/S_). In both cases, product yield was maximized at a C/N ratio of 83.33 g glucose/g NaNO_3_, indicating that, although CBL production by *U. maydis* DSM 4500 is heavily dependent on biomass formation, the efficiency of CBL production could be increased by decreasing the concentration of nitrogen in the growth medium up to a certain point. Therefore, it was concluded that the C/N ratio affected CBL production in two ways. First, at lower C/N ratios, biomass formation was favored. This led to the formation of more viable cells capable of producing CBLs, ultimately leading to a higher CBL titer. On the other hand, product yield per biomass produced and glucose consumed could be increased by increasing the C/N ratio in the growth medium, indicating that CBL production is more efficient under nitrogen-limiting conditions. This indicates the potential of implementing more advanced production protocols in which biomass formation is maximized before shifting conditions toward those which favors the production of CBLs. Product titer was identified as a more important performance indicator and, consequently, the effect of the next important operating condition, the pH of the production medium, was investigated at a C/N ratio of 16.67 g glucose/g NaNO_3_.

#### The effect of pH on the production of CBLs

**Fig. 5:**
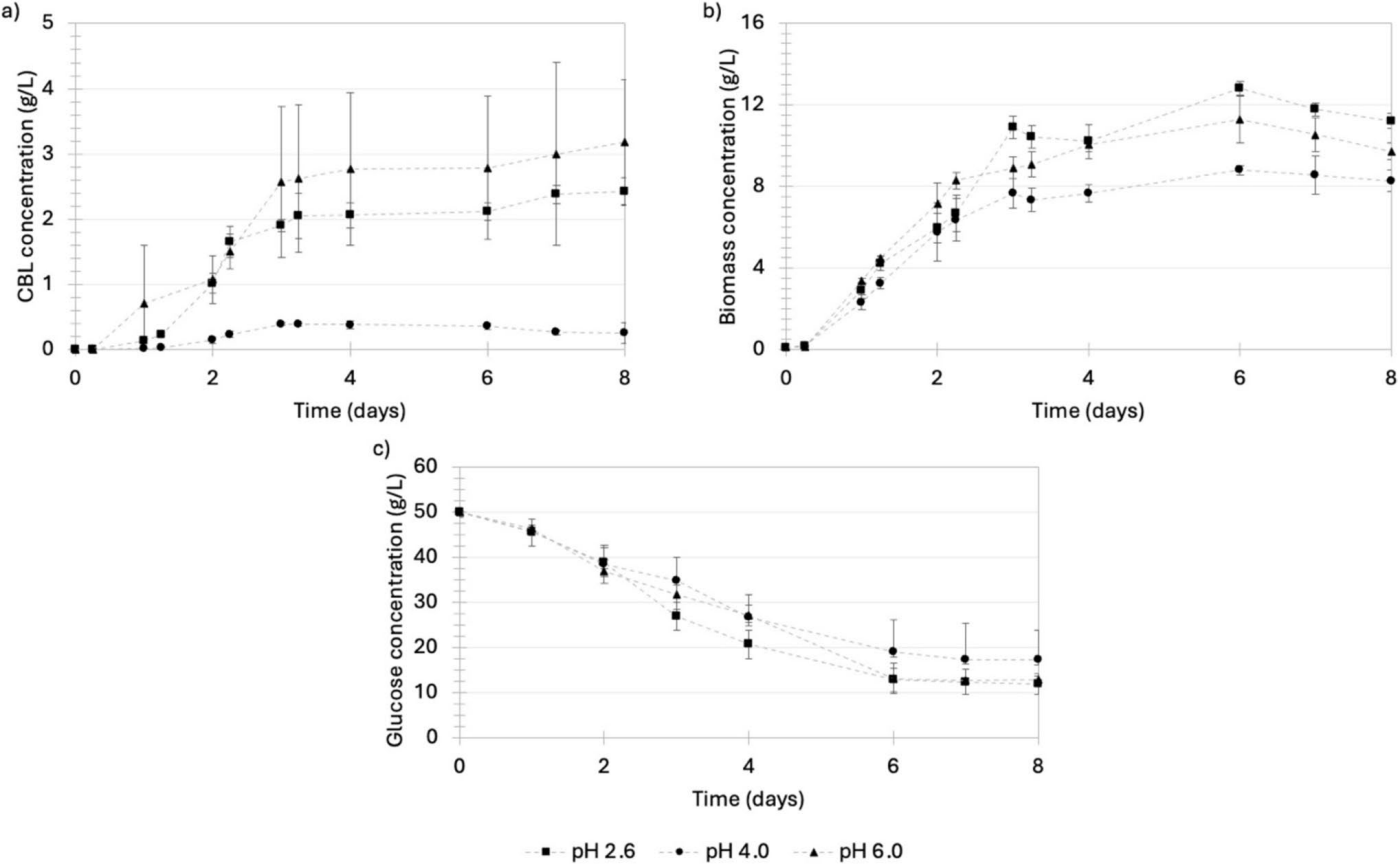
**a** The CBL production, **b** microbial growth, and **c** glucose consumption by *U. maydis* DSM 4500 at a pH of 2.6, 4.0 and 6.0. The C/N ratio in the production medium was 16.67 g glucose/g NaNO_3_. The error bars represent the standard deviation of triplicate replicates

**Fig. 6:**
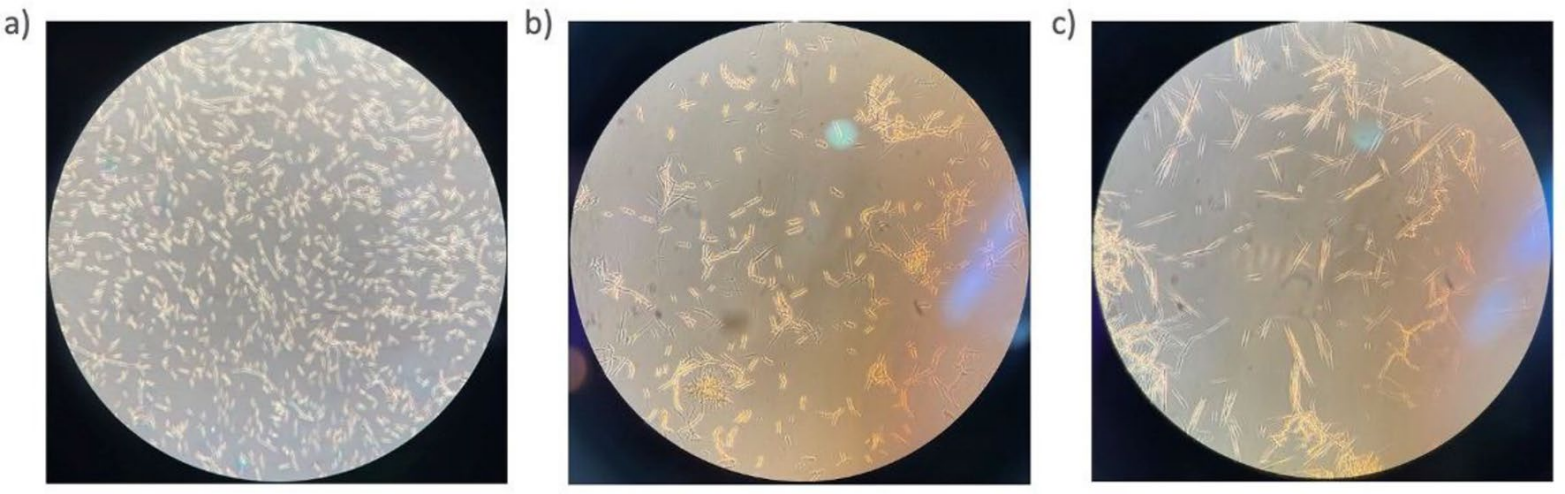
Microscopic images (40 × magnification) of the increased precipitation of CBLs as needle-like crystals when the pH of the culture was decreased from **a** 6.0 to **b** 4.0 and **c** 2.6

Growth curves illustrating the production of CBLs and growth of *U. maydis* DSM 4500 at pH 6.0, 4.0, and 2.6 are shown in Fig. 5. The highest product titer of 3.18 g/L was achieved at pH 6.0, and the second highest product titer of 2.43 g/L was achieved at a pH of 2.6. The lowest product titer of 0.248 g/L was achieved at a pH of 4.0. When these results were considered in conjunction with the product yield per biomass produced and glucose consumed, presented in Table 3, it is clear that the *U. maydis* DSM 4500 performed the best at a pH of 6.0. However, both biomass formation and CBL production were inhibited when the pH was decreased to 4.0, as shown in Fig. 5a, b. CBLs have been shown to be strong antifungal agents and it has been shown that the antifungal effects of CBLs are enhanced when the pH is decreased from 6.0 to 4.0, as shown by Kulakovskaya et al. [33] and Mimee et al. [34]. Therefore, the decrease in biomass concentrations and CBL titers when the pH was decreased from 6.0 to 4.0 was likely due to an increased product inhibitory effect arising from the improved antifungal activity of CBLs at a pH of 4.0 [33, 34]. Interestingly, when the pH of the medium was decreased further, to a pH of 2.6, both biomass formation and CBL production increased. This was likely due to the increased precipitation of CBLs out of solution under these conditions, shown in Fig. 6. The precipitation of CBLs out of the medium led to a decreased concentration of CBLs dissolved in the culture medium. Ultimately, this led to a decreased product inhibitory effect due to the lower concentration of CBLs, along with their antifungal activity, dissolved in solution.

**Table 3:** The average biomass and CBL titers, yield and rates of formation by *U. maydis* DSM 4500 at a C/N ratio of 16.67 g glucose/g NaNO_3_, and a pH of 2.6, 4.0, and 6.0

pH	Titres (g/L)		Yield (g/g)					Rates (g/L.h)	
	Biomass	CBLs	Biomass per glucose consumed	Biomass per nitrogen consumed	CBLs per glucose consumed	CBLs per nitrogen consumed	Product per biomass	Biomass	CBLs
pH 2.6	11.212	2.426	0.295	3.737	0.064	0.809	0.216	0.058	0.013
pH 4.0	8.275	0.249	0.253	2.758	0.008	0.083	0.030	0.043	0.001
pH 6.0	9.717	3.177	0.262	3.239	0.086	1.059	0.327	0.051	0.017

#### The effect of metal ions on the production of CBLs

**Fig. 7:**
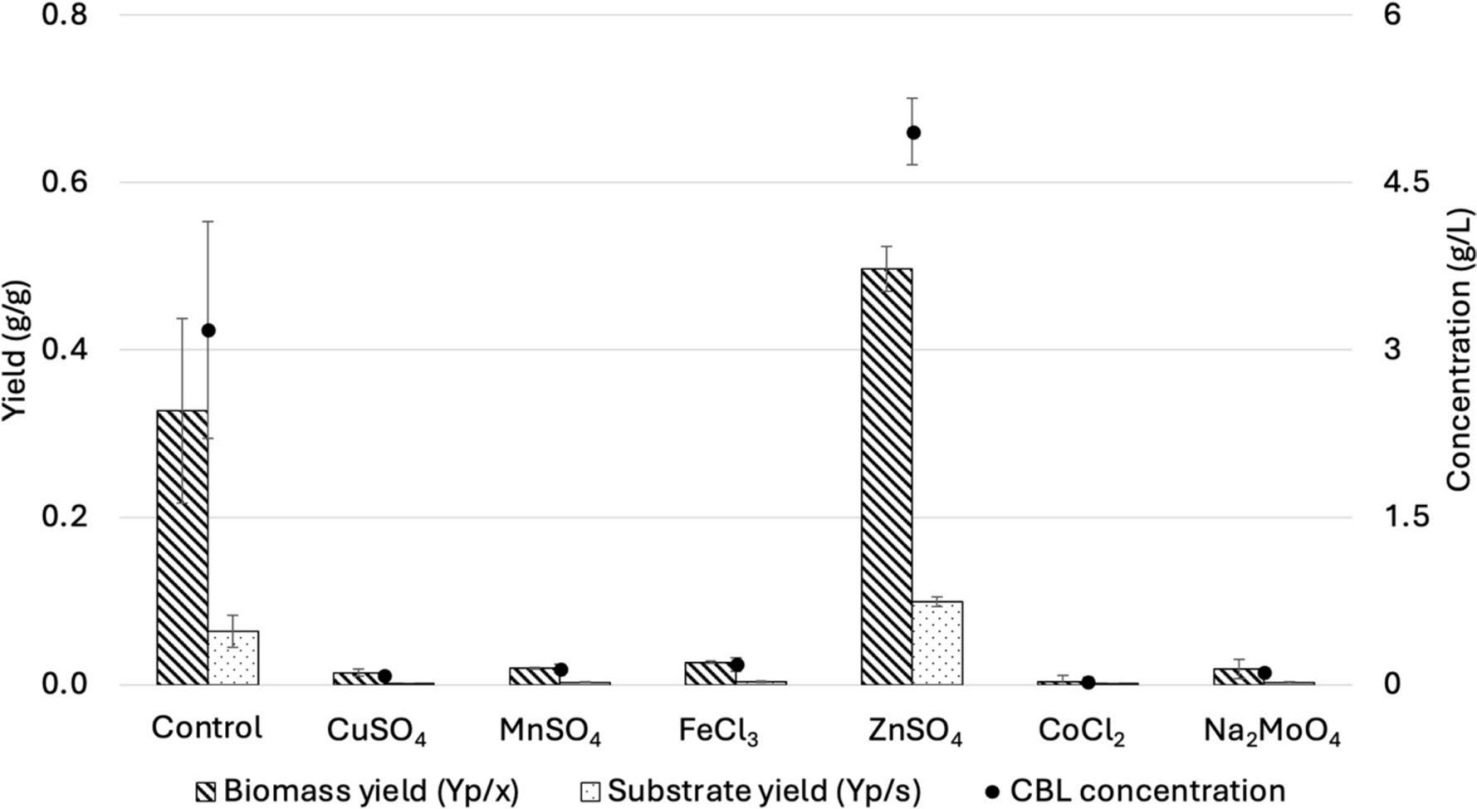
The effect of different trace elements on the production of CBLs by *U. maydis* DSM 4500. The error bars represent the standard deviation of triplicate replicates

To further increase CBL production, the effect of supplementing the cultures with different trace elements on the production of CBLs by *U. maydis* DSM 4500 was investigated, as shown in Fig. 7. All of the different trace elements, except for Zn^2+^, significantly inhibited the production of CBLs. Adding Zn^2+^ at a concentration of 0.04 mg/L increased the CBL titer from 3.18 g/L to 4.95 g/L, representing an increase of 55.66%. When Teichmann et al. [22] sequenced the genes involved in the production of CBLs by *U. maydis*, it was observed that a zinc finger transcription factor Rua1 was involved in regulating CBL synthesis. Zinc finger transcription factors have been shown to consist of nine 30 amino acid units, which are stabilized by zinc ions [35]. This explains why the addition of Zn^2+^ significantly enhanced the production of CBLs.

## Conclusions

This paper presented a process for the production and high-level purification of CBLs by *U. maydis* DSM 4500. After analyzing the CBLs produced by this strain with ESI–MS and NMR, the structure of the main variant was confirmed as 2-O-3-hydroxyoctanoil-3-O-acetyl-β-D-glucopyranosyl-(1–4)-6-O-acetyl-β-D-glucopyranosyl-(1–16)-3,15,16-trihydroxyhexodecanoic acid, commonly referred to as CBL-B. At a C/N ratio of 83.33 g glucose/g NaNO_3_ and a pH of 2.6, *U. maydis* DSM 4500 achieved a CBL titer of 1.36 g/L after 8 days. The focus of the study was then shifted toward understanding the effect of C/N ratio and pH on the production of CBLs by this strain. It was observed that the CBL titer could be increased to 2.43 g/L when the C/N ratio was decreased to 16.67 g glucose/g NaNO_3_. These results indicated that, as opposed to other glycolipids, including MELs, the production of CBLs is less dependent on nitrogen starvation and more closely related to biomass formation. On the other hand, it was observed that the CBL titer could be further increased to 3.18 g/L when the pH of the medium was increased to 6.0. These results demonstrated that *U. maydis* produces CBLs more effectively under neutral conditions, whereas other smut fungi, such as *S. scitamineum*, produced CBLs more effectively under acidic conditions, as demonstrated by Oraby et al. [17]. Interestingly, CBL production was inhibited at a pH of 4.0, likely due to a stronger product inhibitory effect induced by the antifungal CBLs. Finally, it was observed that supplementing the culture with ZnSO_4_ (0.04 mg/L), the CBL titer increased to 4.95 g/L, representing a 55.66% increase compared to the titer achieved at the optimized C/N ratio and pH. Therefore, while providing researchers with a reliable route toward the production of CBLs for future studies, this study also provided a significantly improved understanding of the factors affecting the production of these compounds by *U. maydis* DSM 4500.

## Supplementary Information

Below is the link to the electronic supplementary material.

Supplementary file1 (DOCX 1242 KB)

## Data Availability

Data will be made available on request.
