# Supplementary material for: Bioprocess development for microbial production and purification of cellobiose lipids by the smut fungus Ustilago maydis DSM 4500
Source: Bioprocess Biosyst Eng. 2025 Jan 10;48(3):509–20. doi: 10.1007/s00449-025-03127-3 (PMC11865158; doi:10.1007/s00449-025-03127-3)
Supplement: Supplementary file 1 — Supplementary file1 (DOCX 1242 KB) [file 449_2025_3127_MOESM1_ESM.docx]

# Supplementary sheet


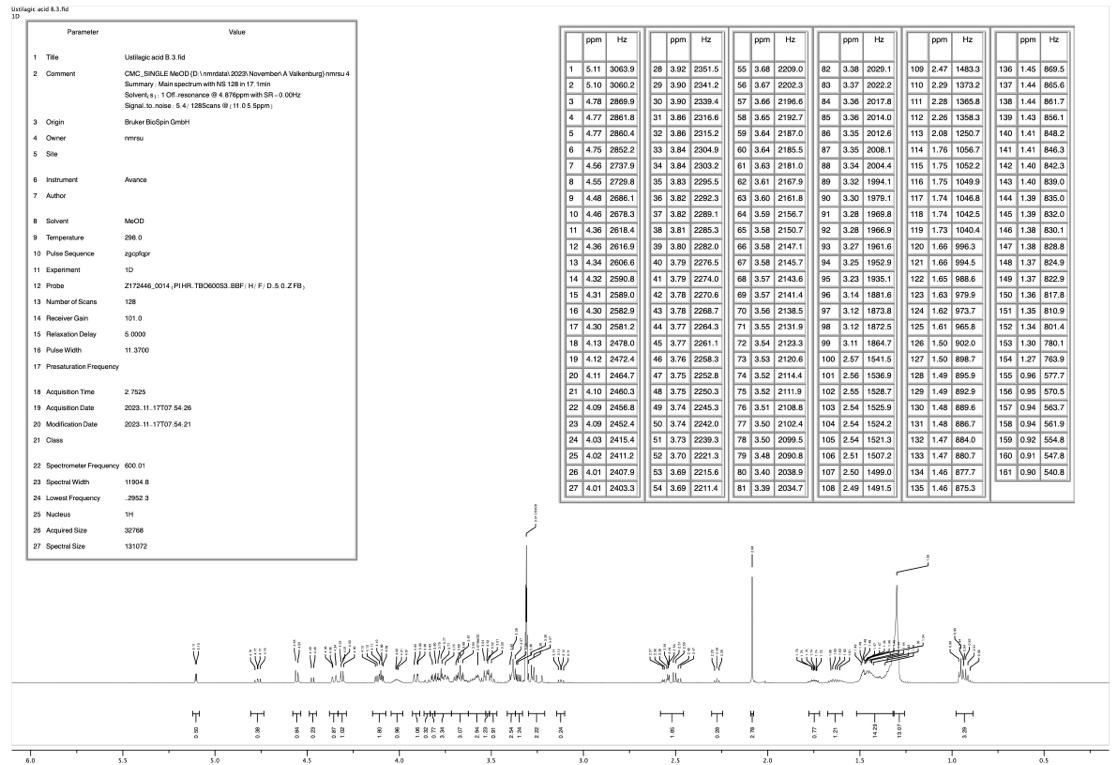


***Fig. 8:*** *The ^1^H NMR spectra of the CBL extract which was purified on TLC plates*

***Fig. 9:*** *The ^13^C NMR spectra of the CBL extract which was purified on TLC plates*


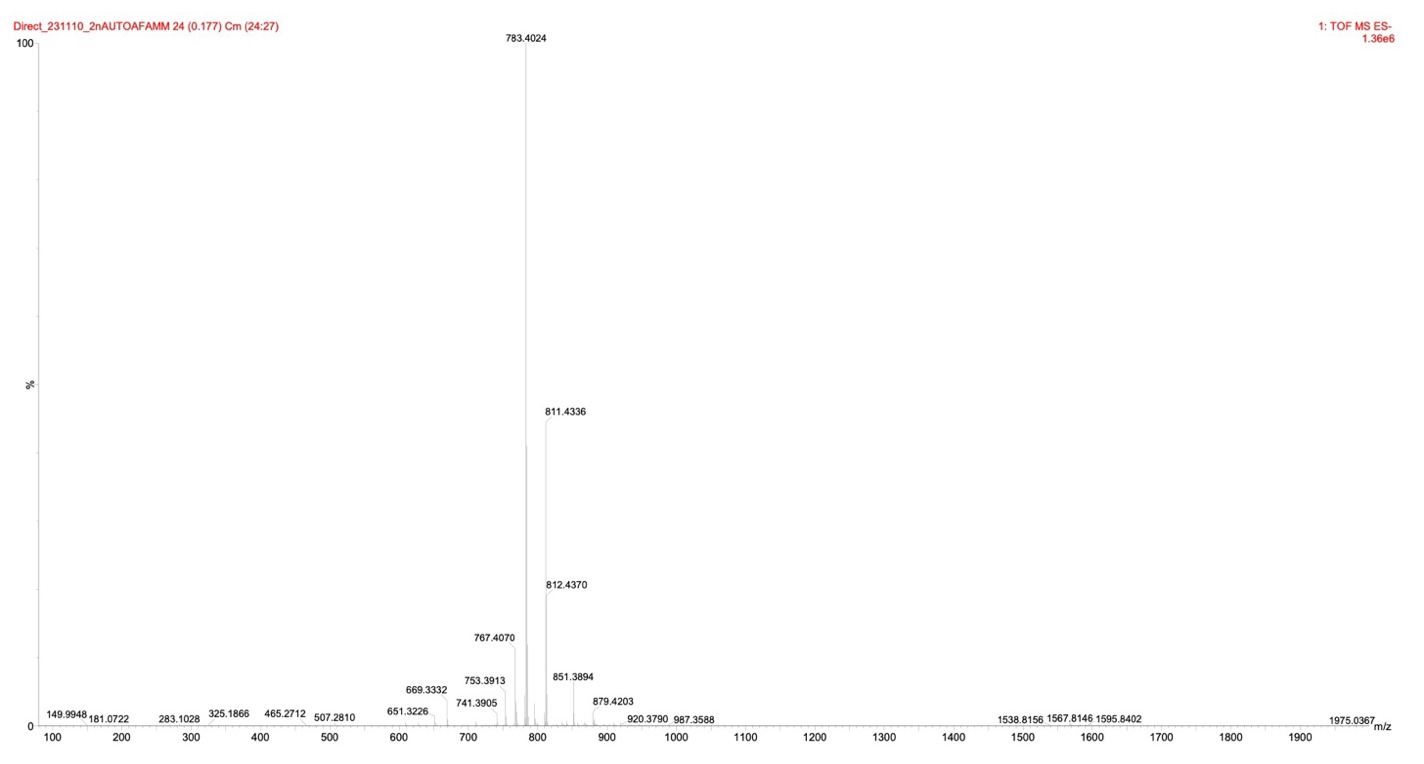


***Fig. 10:*** *The ESI–MS spectra of the CBL extract which was purified on TLC plates*
